# “Growing” meconium cyst in one of a discordant twin: a case report

**DOI:** 10.1186/1757-1626-2-7008

**Published:** 2009-05-14

**Authors:** Adekunle Y Abdulkadir, Lukman O Abdur-Rahman

**Affiliations:** 1 Department of Radiology, University of Ilorin Teaching HospitalIlorinKwara State Nigeria; 2 Department of Paediatrics Surgery, University of Ilorin Teaching HospitalIlorinKwara State Nigeria

## Abstract

**Introduction:**

Giant meconium cyst resulting from antenatal bowel perforation is rare and hardly reported in multiple gestations. We found only four documented cases in the electronic literature.

**Case presentation:**

We report a giant meconium cyst in an 11-hour-old Nigerian boy of a discordant twin having ultrasonographic and plain radiographic diagnosis and surgical confirmation. Increasing abdominal girth from 35 cm to 41 cm within four hours of admission without ascites, pneumoperitneum and significant bowel distension make us assumed the meconium cyst to be “growing” in size. We reviewed the literature and proposed that where the fibrinous wall of meconium cyst allows for distensibility and the communication between the perforated bowel and meconium cyst persists, the meconium cyst acting as reservoir may continue to “grow” in size without remarkable intestinal distension probably, until the elastic limit is lost.

**Conclusion:**

Meconium cyst can occur in twin pregnancy, grow to occupy the abdomen almost completely and may cause neonatal apnoea. The outcome is good post surgery.

## Introduction

Meconium cyst results when meconium leaked into the peritoneum from antenatal perforation of the bowel is walled-off by fibrinous adhesion that ultimately becomes a well-defined capsule of granulation tissue if the perforation remains open [1-4]. The extrusion of meconium into the peritoneal cavity causes sterile chemical peritonitis. This is rare occurrence in about 1: 35000 live births and its progression to form meconium cyst is rarer [1,2-4].

The advent of ultrasound has resulted in the diagnoses of many cases prenatally and in the early neonatal period [7,8]. Prior to this, the diagnosis has been based on the characteristic plain abdominal radiographic findings [3,6]. Magnetic resonance imaging (MRI) is valuable methods that can characterize the lesion and demonstrate its anatomical relationship to the adjacent organs [7]. However, cost and availability is the main draw back to the use of MRI.

A giant meconium cyst diagnosed on postnatal ultrasonography and plain abdominal radiograph in one of a discordant twin is reported because most of the reported cases of either meconium peritonitis or meconium cyst have been in singleton fetus. We found only four cases documented in association with multiple pregnancies in the electronic literature [8-10].

## Case presentation

An 11-hour-old Nigerian boyof Yoruba ethnicity, first of a discordant twin delivered at home by a 27-year-old woman, para 3 ^+ 0^, 4 alive, under the supervision of a community nurse, was brought to our Hospital because of a progressively increasing abdominal swelling. Prior to presentation, he had two episodes of non-bilious and non-projectile vomiting. Antenatal care was supervised at a community clinic. Delivery was at 37 weeks 6 days menstrual age and the labour lasted for about 8 hours. Both babies cried spontaneously at birth and required no resuscitation. However, the boy had increasing abdominal distension overtime, vomited on attempted feeding and soon begins to have apnoea. These were the reasons for his referral to our centre.

At presentation, he was dyspoeic (60 cycles per min.), tachycardic (140 beats per min.) and hypothermic (35 °C). The abdomen was grossly distended, tensed and shining. Umbilical cord was tied with thread but appear clean. Bowel sounds were hypoactive. The abdominal girth increased from 35 cm to 41 cm within four hours of admission. The head circumference was 31.3 cm. The chest was clinically clear. He weighed 2.5 kg while the second twin weighs 2.7 kg.

Laboratory work ups, which included full blood count, blood culture and serum chemistry were all within normal range.

**Figure 1.**
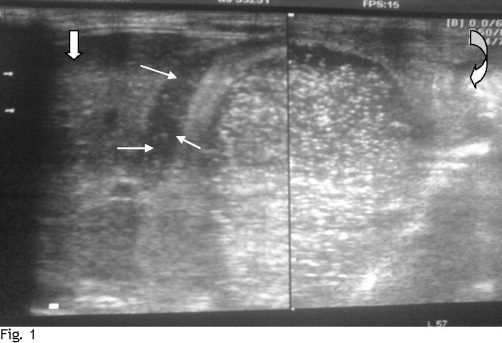
Transabdominal sonograms showing a well-circumscribed giant meconium cyst. Note the multiple brightly echogenic internal echoes and layering (image to the left). The deuodenum (short arrows) and liver (block arrow) are normal. The compressed urinary bladder is indicated by curved arrow.

**Figure 2.**
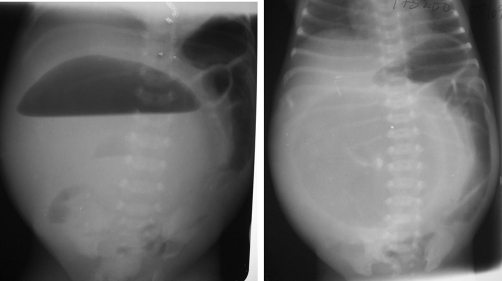
Plain abdominal radiographs (erect and supine) of an 11-hour-old boy with giant meconium cyst. Note the giant central mass with air-fluid level (left image), the peripherally displaced bowel loops with no remarkable distension, the elevated diaphragms to the level of 6th rib posteriorly and the splaying of the ribs.

Abdominal ultrasound showed a thick-walled cystic mass occupying nearly the entire abdomen. It has brightly echogenic internal echoes, layering and calcifications (Figure 1). The mass displaces the liver into the chest. The bowel loops were peripherally displaced and contains excess gas. Their calibers were sonographically normal. The small bowel loops were not more than 27 mm at any point where accessible for measurement. There was no ascites. Based on these sonographic findings, meconium cyst was suspected. The plain abdominal radiographic findings, of fluid level in a giant central lucency having a circumscribed thick-rim opacity, internal calcifications and peripherally displacing bowel loops was complimentary (Figure 2). The ribs were splayed and both hemidiaphragms were elevated to the level of the 6^th^ rib posteriorly.

**Figure 3.**
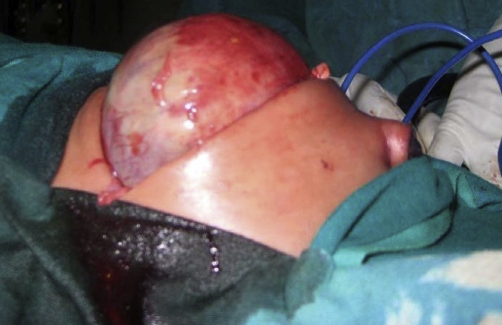
Intraoperative photograph of the baby showing the giant meconium cyst.

Exploratory laparotomy revealed a giant meconium cyst (Figure 3), terminal ileal atresia with defective mesentry (type IIIa atresia) and there was about 4cm blind stump of distal ileum attached to the colon. The treatment options were discussed with the family and it was felt better to proceed to a bowel resection with ileocolic anastomosis. The operation was successful and the patient did well postoperatively and was discharged five days later.

## Discussion

Antenatal perforation of the bowel leading to meconium peritonitis occasionally occurs as a localized, encysted, collection of meconium ranging from a few centimeters in size to huge cysts occupying most of the abdominal cavity when the perforation remains open [1,4]. Fibro-adhesive meconium peritonitis usually results if the perforation closes spontaneously before birth. When the perforation remains open, meconium cyst communicating with the perforated bowel site results [1,4]. Supporting this was the intraoperative finding of a giant cyst containing meconium surrounding the terminal ileum, the site of perforation in our case. This phenomenon is rare in multiple pregnancies. Whether this is a true rarity or under reporting is unknown. Again, the sex incidence of meconium peritonitis is uncertain. In contrast to Tseng et al [5] series involving 19 cases of meconium peritonitis having 6 boys and 13 girls, Ali and ul-Hassan [11] series of 20 meconium peritonitis have 14 boys and 6 girls while Hsu et al [12] series, also involving 20 meconium peritonitis have male female ratio of 1.5:1.

The rapid abdominal distension in our patient post delivery, which culminated into respiratory distress presumably due to diaphragmatic splinting from raised intra-abdominal pressure, necessitated urgent and minimally non-invasive diagnostic radiological work-up with ultrasound and plain radiograph of the abdomen prior to emergency surgery.

Plain radiograph is of good diagnostic capability. The radiographic findings may include abdominal distension, intestinal dilatation, pneumoperitoneum, air-fluid levels and calcifications [2,4,6]. The giant central lucency with circumscribing thick rim opacity, internal calcifications and a giant single air-fluid level on the erect abdominal radiograph of our patient, better described meconiun cyst. However, antenatal volvulus, communicating duplication cyst, large Meckel's diverticulum, hydrocolpos with fistula and congenital pouch colon of anorectal malformations, all may resemble gas-containing meconium cysts, which commonly calcify [2,3,5,6]. Tseng et al [5] demonstrated calcification in about 80% of meconium peritonitis. Similarly, Pan et al [3] reported that meconium peritonitis is one of the few conditions that can be diagnosed before birth and is almost the only condition around the time of birth to produce calcification in the abdomen. However, there is the need for caution as intra-luminal meconium calcifications are a rare cause of neonatal abdominal calcifications and may easily be misinterpreted as meconium peritonitis [6]. Miller et al [6] reported three cases of intra-luminal calcifications in anorectal malformation associated with rectourethral fistula, which they assumed intestinal stasis and mixing of urine with meconium as the predisposing factors for such calcifications. Discrete punctate flecks of calcifications within the distribution of the bowel is characteristic of intra-luminal calcifications, unlike in meconium peritonitis, where the calcifications are linear and plaque-like, occurring anywhere in the abdominal cavity and scrotum [3].

The lucency (gas) within the meconium cyst in this case indicates persistent communication between the perforated bowel and the cyst cavity and was confirmed at surgery. The increasing abdominal distension and respiratory distress in our patient with no commensurate radiographic, ultrasonic and intraoperative findings of bowel distension make us assume a “growing” meconium cyst. Probably where the fibrinous wall of meconium cyst allows for distensibility, proximal bowel dilatation may not be remarkable rather the meconium cyst acting as reservoir may continue, “Growing” in size. We assume that in a “growing” meconium cyst, the site of perforation must act as a one-way valve that allows intestinal emptying without refluxing. Emptying into the cyst may persist until the emptying pressure is exceeded before remarkable intestinal distension can occur. Further study is required to corroborate or refutes our arguments. However, some cases having no bowel distension have been documented [11,12].

The location of the bowel perforation in our case at the pre atretic segment of the ileum is similar to few other reported cases [1,5]. The increasing size of the cyst resulted in the displacement of the proximal bowel loops to the left flank and superiorly. The giant cyst in this case occupies virtually the entire peritoneal cavity (Figure 2a & b). Thus, the radiographic findings in meconium cyst may depend on the size of the cyst or persistence perforation after birth or not. Review of several authors reports showed that air-fluid level extending almost completely across the peritoneal cavity, with the intestines displaced peripherally, with or without calcifications as in our case are characteristic of a giant meconiun cyst [2,7]. If the perforation has sealed spontaneously in utero, the cyst remains free of gas and no surgical intervention will be necessary [1,4]. However, surgery is required for all case of meconium cysts and the outcome as in our patient is generally good [1,2,5]

## Conclusion

Meconium cyst can occur in twin pregnancy, grow to occupy the abdomen almost completely and may cause neonatal apnoea. Ultrasonography and plain radiography are invaluable in the diagnosis. We propose a possible explanation for the development of giant meconium cyst, which we have tag a “growing” meconium cyst.
